# SleepSync: Early Testing of a Personalised Sleep–Wake Management Smartphone Application for Improving Sleep and Cognitive Fitness in Defence Shift Workers

**DOI:** 10.3390/clockssleep6020019

**Published:** 2024-05-29

**Authors:** Prerna Varma, Svetlana Postnova, Stuart Knock, Mark E. Howard, Eugene Aidman, Shantha W. M. Rajaratnam, Tracey L. Sletten

**Affiliations:** 1 Turner Institute for Brain and Mental Health and School of Psychological Sciences, Monash University, Clayton, VIC 3168, Australia; 2 School of Physics, The University of Sydney, Camperdown, NSW 2050, Australia; 3 Institute for Breathing and Sleep, Austin Health, Heidelberg, VIC 3084, Australia; 4 Faculty of Medicine, Dentistry and Health Sciences, University of Melbourne, Parkville, VIC 3010, Australia; 5 Defence, Science and Technology Group, Department of Defence, Edinburgh, SA 5111, Australia; eugene.aidman@defence.gov.au; 6 School of Biomedical Sciences & Pharmacy, University of Newcastle, Callaghan, NSW 2308, Australia; 7 Division of Sleep and Circadian Disorders, Departments of Medicine and Neurology, Brigham and Women’s Hospital, Boston, MA 02115, USA; 8 Division of Sleep Medicine, Harvard Medical School, Boston, MA 02115, USA

**Keywords:** digital health, shift work, sleep health support, military health, behaviour change, intervention development

## Abstract

Shift work, long work hours, and operational tasks contribute to sleep and circadian disruption in defence personnel, with profound impacts on cognition. To address this, a digital technology, the SleepSync app, was designed for use in defence. A pre-post design study was undertaken to examine whether four weeks app use improved sleep and cognitive fitness (high performance neurocognition) in a cohort of shift workers from the Royal Australian Air Force. In total, 13 of approximately 20 shift-working personnel from one base volunteered for the study. Sleep outcomes were assessed using the Insomnia Severity Index (ISI), the Patient-Reported Outcomes Measurement Information System (PROMIS), Sleep Disturbance and Sleep-Related Impairment Scales, the Glasgow Sleep Effort Scale, the Sleep Hygiene Index, and mental health was assessed using the Depression, Anxiety, and Stress Scale-21. Sustained attention was measured using the 3-min Psychomotor Vigilance Task (PVT) and controlled response using the NBack. Results showed significant improvements in insomnia (ISI scores 10.31 at baseline and 7.50 after app use), sleep-related impairments (SRI T-scores 53.03 at baseline to 46.75 post-app use), and healthy sleep practices (SHI scores 21.61 at baseline to 18.83 post-app use; all *p* < 0.001). Trends for improvement were recorded for depression. NBack incorrect responses reduced significantly (9.36 at baseline; reduced by −3.87 at last week of app use, *p* < 0.001), but no other objective measures improved. These findings suggest that SleepSync may improve sleep and positively enhance cognitive fitness but warrants further investigation in large samples. Randomised control trials with other cohorts of defence personnel are needed to confirm the utility of this intervention in defence settings.

## 1. Introduction

Defence personnel often work in safety-critical roles, undertaking shift work in conditions of high stress and high psychophysiological demand. Shift work, long work hours, and operational tasks contribute to chronic disruption of sleep and circadian rhythms (i.e., a mismatch between internal biological clock and external environmental cues) [1,2,3] among defence personnel, which can adversely affect cognition [4,5]. Insomnia is prevalent in up to 63% of active duty members and veterans and is one of the most common reasons for mental health referrals [6,7]. Diagnosed sleep disorders in active-duty personnel can increase the risk of motor vehicle accidents, and work related injuries [8,9]. Disruptions to sleep and circadian rhythms in the military are also linked to impairments in higher level cognitive functioning, compromising work readiness and potentially decreasing performance on tasks that require rapid decision-making [3,10]. Given the profound impacts of sleep on operational efficacy, safety and performance, sleep-wake management is recognised as a key target for promoting high-performance neurocognition or “cognitive fitness” in defence personnel. Cognitive fitness is an integrative framework that focuses on the capacity to train and deploy neurocognitive resources, such as self-awareness, attention, co-action, task resilience, and sleep recovery to meet or exceed the demands of operational task performance [11].

Current approaches towards sleep–wake management in shift work are systemic, including shift scheduling, workplace lighting, napping, and broad fatigue management programs [4,5,12,13,14]. While these interventions have some demonstrated success, they are inconsistently applied across shift-working industries and often do not account for unpredictable variability in shift rosters, or personal or social commitments. Non-pharmacological, psychoeducation-based approaches such as stress reduction, timed caffeine, exercise, diet, and healthy sleep practices are aimed to equip workforces with resources they can implement themselves [15,16,17]. However, it may be challenging for individuals to determine timing or dosage of interventions, such as the timing of sleep to optimise alertness during shifts, which may prevent individuals from implementing these successfully. It is also difficult to provide individual timing for these interventions without information about individual shift schedules, lifestyles, or habitual sleep practices. Existing digital technologies and smartphone applications are geared towards night sleepers and do not necessarily offer individualised support for shift workers. To address this, digital and manual approaches utilising circadian biology [18,19,20,21] have been trialled to automatically deliver sleep–wake recommendations personalised to the user’s shift schedules and personal commitments [13,15,22,23]. Initial testing shows that these interventions have positive impacts on insomnia, but whether they have any effect on other domains of functioning or cognitive fitness warrants further investigation.

This study aimed to examine the early efficacy of one such intervention, the SleepSync app [13] in improving the following domains of cognitive fitness [11,24]: sleep, mental health, sustained attention and controlled response in a cohort of shift-working defence personnel in the Australian Defence Force.

## 2. Results

Participant characteristics are presented in Table 1. In total, 18 individuals expressed interest to participate in the study (of approximately 20 shift workers on the base). Five participants were unable to complete due to health or travel reasons. The final dataset included 13 participants. Job role is not included in participant characteristics to maintain confidentiality. All participants were undertaking full-time shift work during the study. While nine participants reported undertaking night shifts prior to the start of the study, only six participants completed night shifts during the study, while the rest of the participants worked morning and evening shifts. On average, participants logged onto the app on 37 out of 42 study days (±4 days). During the trial, 1123 recommendation requests were sent to the Alertness API, equating to 86 requests for new or updated recommendations for each participant. When asked to comment on the likelihood of using recommendations from the app in daily life post-trial, 9 out of 13 participants responded that were “likely” or “very likely” to use them; 11 out of 13 participants reported that they found other recommendations related to caffeine use and educational toolkit for sleep “somewhat” to “very” helpful.

**Figure 1:**
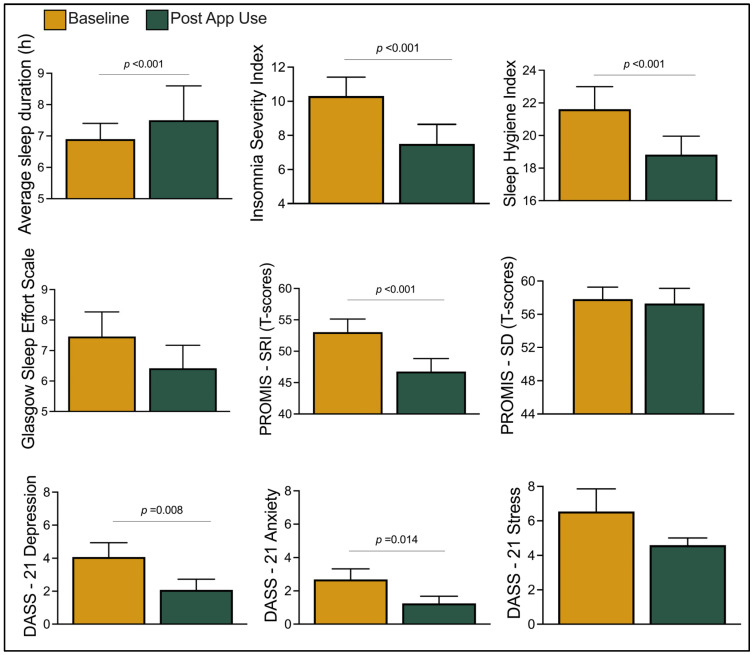
Changes from baseline to post-app use for average sleep duration; Insomnia Severity Index (ISI); Sleep Hygiene Index (SHI), PROMIS—Sleep Related Impairments (SRI); Sleep Disturbance (SD); and Depression, Stress, and Anxiety Scale-21 (DASS-21). Bonferroni correction applied; alpha value set to 0.005. Error bars indicate standard error of mean.

A one-sample Wilcoxon test revealed that multiple domains of sleep improved. Average sleep duration increased by half an hour from baseline to post-app use (Figure 1). At baseline, the average ISI score was 10.31, above the community sample cutoff of 10 to screen for insomnia. These scores improved post-app use (Baseline = 10.31 ± 3.89; Post-app use = 7.50 ± 3.99; *r* = 0.48; *p* < 0.001), indicating a clinically relevant reduction in insomnia. Following similar trends, scores on SHI (Baseline = 21.61 ± 4.81; Post-app use = 18.83 ± 3.71; *r* = 0.45; *p* < 0.001) and PROMIS-SRI (T-score Baseline = 53.03 ± 7.56; T-score Post-app use = 46.75 ± 7.14; *r* = 0.88; *p* < 0.001) significantly improved with moderate and large effect sizes. There were no significant changes in scores on GSES and PROMIS-SD.

Symptoms of adverse mental health were low at baseline and declined further post-app use, particularly for depression (Baseline = 4.07 ± 2.95; Post-app use = 2.08 ± 2.13; *p* = 0.008). However, this difference was not statistically significant upon applying Bonferroni corrections.

For all objective measures, best-fitting models (based on AIC) included testing period and shift type with all models, explaining 23% to 47% variance in scores for PVT outcomes and 30% to 33% for NBack outcomes. Significant inter-individual variability was observed for all objective outcomes, with EDFs for all models greater than one.

For PVT, generalised additive mixed models revealed that relative to baseline, there was modest worsening of the slowest 10% reciprocal RT (Intercept = 2.47, Week 6 of App Use = −33; CI = −0.53–−0.14, *p* = 0.003) and mean RT (Intercept = 274.86, Week 6 of App Use = 35.89; CI = 10.30–61.48, *p* = 0.006) after four weeks of app intervention (Table 2). There was a nonsignificant trend for improvement in the fastest 10% RT (Intercept = 211.72, Week 6 of App Use = −7.45; CI = −14.46–−0.44, *p* = 0.03) and number of lapses (Intercept = 4.98, Week 6 of App Use = −1.06; CI = −2.27–0.16, *p* = 0.08). No changes were observed for false starts or mean reciprocal RT.

There was a significant reduction in NBack incorrect responses at four weeks of app intervention (Intercept = 9.36, Week 6 of App Use = −3.87; CI = −5.49–−2.24, *p* < 0.001). However, no improvements were recorded for NBack mean RT, number of correct responses, or accuracy scores post-app use (Table 3). No significant differences were revealed for any outcomes based on type of shift or whether the tests were conducted at the start or the end of the shift.

## 3. Discussion

This study examined the efficacy of the SleepSync app in improving multiple domains of sleep and cognitive fitness in a cohort of defence personnel in a real-world setting. For the first time, a digital technology was designed and subsequently, used with an intention to aid cognitive fitness in military settings. Sleep timing recommendations were delivered using well validated biomathematical models that adapted to changing rosters, personal commitments, and sleep needs. Four weeks of use of the SleepSync app was associated with improvements in multiple domains of cognitive fitness, including sleep, daily functioning, as well as modest changes in objective metrics. While participants did not report clinically significant symptoms of adverse mental health, there were discernible trends for positive changes in mental health as well, particularly depression. These findings add to an emerging body of evidence for the use of personalised sleep recommendations to improve sleep and overall health in shift workers [13,15,22,23].

There were marked changes in sleep post-app use, with self-reported average sleep duration increasing by 30 min. There was clinically relevant reduction in symptoms of insomnia, with average scores no longer qualifying for the cut-off used to screen for insomnia. Functional impairments related to sleep (as measured by PROMIS SRI), such as reduced alertness, increased sleepiness and tiredness during waking hours improved. Participants recorded less behaviours that can compromise sleep, particularly for SHI items related to not “*staying in bed longer I should*”, not “*doing something that may wake me up before bedtime*”, and not “*think, plan, or worry when I am in bed*”. Personalised sleep recommendations, along with the implementation of healthy sleep practices suggested by the app, may have contributed to these positive changes in sleep. The findings are also encouraging, as shift workers may not have awareness or may find it difficult to engage in sleep hygiene practices due to variability in shift schedules or personal circumstances and because poor sleep hygiene is one of the key risk factors for experiencing shift work disorder [25,26]. While these results require validation across larger trials, tailored support to implement healthy sleep practices can continue to be viable target of intervention to improve sleep in shift workers [17].

There were varying findings for sustained attention and controlled response upon the use of the app. For PVT, there was a trend for improvement in fastest mean RT but modest worsening in mean RT and slowest 10% RT. For NBack, the number of incorrect responses reduced at four weeks of app use, but other outcomes did not improve. These findings being somewhat contradictory may be explained by several factors, including a limited sample size which may not be enough power to detect significant or clinically relevant changes in all metrics of cognitive fitness. Quite importantly, the study duration may have led to positive changes in behaviour but may not have been long enough to translate into more tangible changes in cognitive fitness. While there are no guidelines on the length of intervention for improving performance, research in older adults shows that cognitive training for anywhere from one year to five years can have benefits for functional outcomes [27], while sustained behaviour change may take around 10 weeks [28]. Anecdotally, participants reported boredom at completing the tests and suggested that they were long and tedious to complete during shift (approximately ten minutes), which was a deterrent to their motivation. Given that sleep is critical in maintaining optimal executive functioning [29,30], long-term deployment and testing of the app—along with shorter, more practical measures of performance—may be required to assess cognitive fitness in safety-critical operations.

Our study showed significant individual variability in performance for both sustained attention and controlled response. While expected, this finding reiterates the need for tailored support for defence personnel to improve sleep and increase operational readiness. Previous research has shown that this individual variability, particularly for sustained attention can be traced to key group of factors: environmental and behavioural influences, such as shift schedules, tasks, daily sleep, behavioural and lifestyle choices; and individual’s endogenous physiological parameters, such as chronotype and circadian timing, sensitivity, and response to light [31,32,33,34,35]. While this study accounted for variability in environmental and behavioural influences, through calendar integration, allowing participants to enter their shifts and other commitments, as well as habitual and daily sleep, it did not account for variability in their circadian timing. Given that circadian timing can vary by up five hours in day workers, even in controlled settings [36,37], accurate prediction of circadian timing is required for recommending appropriate timing for sleep and other countermeasures [36]. Biomathematical models can provide predictions of circadian timing following inputs related to chronotype, sleep, and light exposure, among other factors [36,37], and can thus be used to generate recommendations to optimise sustained attention during and after shifts. Using circadian timing while deploying these models to optimise alertness is a much-needed area for future research.

The current study supports the potential benefits and utility of implementing digital interventions to deliver tailored sleep recommendations for shift-working defence personnel. As an automated, individual-level intervention, the app provides a mechanism to effectively deliver real time advice for shift workers. Unpredictability in work-rest patterns and changes in daily sleep patterns may make it difficult to provide sleep recommendations that are tailored to a shift worker’s needs. A digital technology can address that unpredictability, updating recommendations using biomathematical models whenever a user enters or modifies information. Second, defence personnel and veterans may be reluctant to seek treatment for their concerns due to a potential impact on their career [38]. Shift workers may also find it challenging to seek clinical support for the concerns. Generic sleep interventions, such as cognitive behaviour therapy for insomnia, which is the gold standard for general populations, do not produce clinically significant improvements in shift workers [39]. Previous research has also shown that non-pharmacological and organisational interventions, such as lighting, workplace napping may be beneficial for performance in shift workers, but does not always benefit sleep-related outcomes, demonstrating a need for tailored approaches [39,40]. Digital technologies can provide an accessible, confidential, and tailored pathway to deliver optimal support for sleep, with potential impacts on their health and cognition.

While the study shows positive outcomes for using digital technology to aid cognitive fitness, there are some notable limitations and considerations. The study had a small sample size limited by the available participant pool. Results should be interpreted with this in mind. Larger, randomised control trials with other groups are necessary to establish the effectiveness of SleepSync across different workforces. An extensive systematic review of interventions for physical health and sleep in shift workers [12] observed that for most shift work interventions with a waitlist or a sham control group, the efficacy of intervention was significantly higher than control for improving both objective and subjective sleep outcomes. This suggests that interventions designed specifically for shift work may have benefits beyond the placebo effect. While this study did not compare compliance or differences in recommended sleep–wake and baseline sleep patterns, a previous pilot examining manual use of biomathematical models showed that shift workers’ sleep and wake schedules were aligned with model recommended sleep and wake schedules for at least 70% of the times during a one week period [23].Examining daily compliance can be quite informative, especially in understanding where and who this intervention can benefit the most and where implementation can be improved.

## 4. Materials and Methods

### 4.1. Participants

Thirteen uniformed members of the Royal Australian Air Force (RAAF) aged 22–46 years and employed as air traffic controllers volunteered to participate in this study. The RAAF base selected for the study had approximately twenty shift working officers at any given time primarily working one of the following two shift rotations: (i) two morning shifts (starting between 0545 and 0700 h; ending between 1300 and 1500 h), followed by two evening shifts (starting between 1100 and 1400 h; ending between 1900 and 2200 h) and one night shift (between 2200 and 0600 h); and/or (ii) two morning and two evening shifts. Study participation had received command approval, and participants provided informed consent. The following exclusion criteria were applied:

1. Having an untreated sleep disorder other than insomnia or shift work disorder (such as restless leg syndrome, central or obstructive sleep apnoea, or narcolepsy).
2. Having an untreated medical condition that may impact sleep, such as diabetes, thyroid disease, hypertension, or neurological conditions.
3. Having an untreated mental health (psychiatric) condition that may impact sleep other than depression or anxiety.
4. Current caffeine consumption > 500 mg per day.
5. Alcohol consumption > 20 standard drinks in a week.
6. Transmeridian travel in the past month.
7. History of substance abuse in the past 12 months.

### 4.2. Intervention

The study intervention was the SleepSync app. SleepSync is a personalised sleep–wake management tool which delivers recommendations for sleep timings based on individuals’ rosters, personal commitments, and daily commute times [13]. In addition to recommendations, the app also provides a toolkit with educational resources on managing lifestyle as a shift worker, sleep and circadian rhythms, caffeine use, light exposure, and healthy sleep practices. Upon logging onto the app, users are provided walkthrough of the app and requested to enter information about their habitual sleep patterns, work patterns (either manually or via syncing with one of the commercial calendar services, such as Google, Outlook, iCal, etc.), personal commitments, and daily sleep. The app then uses this data to generate sleep recommendations for a week, including sleep timings and duration of sleep. Users can view trends and summary data related to their sleep over the last 24 h, one week and one month. Previous user testing of the app in healthcare and other shift workers showed high user engagement, with 82% of users finding it easy to integrate the app into their lives and majority reporting that the app had an influence on their daily behaviours [13].

The initial app was codesigned with the healthcare sector and used a decision tree algorithm [13,15], which was then redesigned with Defence (via qualitative, semi-structured interviews). At this stage, the decision tree model was replaced by the Model of Arousal Dynamics. The Model of Arousal Dynamics is a biomathematical model of sleep, alertness, and circadian rhythms that has been specifically validated and calibrated against shift work and circadian misalignment data [18,19]. The Model of Arousal Dynamics includes circadian oscillator and comprises of physiologically based flip-flop switch between sleep active and wake neuronal populations [18,19], which allows for prediction of sleep propensity. By using circadian and homeostatic drives, the model can predict sleepiness and performance outcomes [41] and optimised to provide recommendations that maximise alertness during shifts and on commute or maximise total sleep time obtained within a 24 h window. For SleepSync, recommendations were made using the “predictions” features of the model, which predicts the need for sleep based on historical and current context related to sleep and shifts. The model was deployed using the Application Programming Interface, Alertness API (www.alertnessapi.com), that was used in the backend of the SleepSync app. The theoretical underpinnings related to the selection and testing of biomathematical models and adherence with recommendations are described elsewhere [23]. To use SleepSync, participants were instructed to add their shift work schedules and personal commitments to the app up to four weeks in advance either through integration with their existing calendar or by using the “add shifts” feature of the app. Recommendations were displayed for up to seven days in advance and updated when participants completed sleep diaries on the app or entered and/or modified information related to shifts or personal commitments.

### 4.3. Procedure

The study was an open label, pre-post design approved by the Defence Science and Technology Group Low-Risk Ethics Committee (DSTG LREP Approval ID: LD 05-22) and registered with the Monash University Human Research Ethics Committee (Approval ID: 35256). Recruitment was facilitated through the leadership of the squadron. An advertisement flyer was emailed to the workforce by the commanding officer, with interested individuals advised to contact the research team directly. Consenting participants were provided an access code and a link to install the SleepSync app on their mobile device. Upon downloading, participants received a short tutorial on the app providing information on how to use and access its features.

The first two weeks served as a baseline period, where participants could use the app to enter and record sleep timings but did not receive any recommendations. At the start of the baseline period, participants completed an online survey to collect demographic information. Self-report measures were completed via the Qualtrics platform (Qualtrics, Provo, UT). Objective measures were completed using the iPad-based Joggle Research app, as previously used in military settings (www.joggleresearch.com). A test battery for objective measures was completed at the start and end of the first morning shift, first evening shift and first night shift at baseline, two weeks of app intervention (i.e., the second week of receiving recommendations), and four weeks of app intervention (i.e., the fourth week of receiving recommendations).

### 4.4. Measures

All measures used in the study were determined based on previous research on different domains of cognitive fitness [11,24] and findings from a Delphi consensus study that examined cognitive factors that drive performance in critical occupational settings [42]. The following cognitive fitness domains were assessed: sleep, mental health, alertness, and controlled response.

Average sleep duration was measured as a single item modified from the Pittsburgh Sleep Quality Index (“During the past month, how many hours of actual sleep did you get in a 24-h period (in hours and minutes)”). Insomnia symptoms were examined using the Insomnia Severity Index (ISI) [43], which is a seven-item scale that uses the Diagnostic and Statistical Measure of Mental Disorders symptom criteria to screen for insomnia. The brief screening tool includes questions related to difficulties in falling asleep, staying asleep, or waking up too early; satisfaction with sleep patterns; and sleep disturbances interfering with daily life. Sleep disturbance was assessed using the PROMIS Sleep Disturbance (PROMIS SD) bank [44], which examines restlessness and difficulties related to sleep during a seven-day period. Sleep-related impairments were assessed using the Patient Reported Outcomes Measurement Information System Sleep-Related Impairment (PROMIS SRI) bank [44], which is an eight-item scale that assesses functional impairments associated with sleep, such as tiredness, alertness, and sleepiness. Total scores of both PROMIS SD and PROMIS SRI are converted to T-scores. Sleep effort, which examines persistent preoccupation with sleep, was assessed using the Glasgow Sleep Effort Scale (GSES) [45], which is a seven-item scale that includes questions related to putting effort into sleeping, worrying about not sleeping, anxiety related to bed, and worries related to the consequences of not sleeping enough. The presence of behaviours that may compromise sleep or habits related to sleep was measured using the Sleep Hygiene Index (SHI) [29], which includes items related to use of alcohol, tobacco, and caffeine before bedtime, exercising before bedtime, or performing mentally stimulating activities before bed. Mental health was examined using the Depression, Anxiety, and Stress Scale-21 (DASS-21), which is a common screening tool for adverse mental health symptoms.

Sustained attention was assessed using the three-minute Psychomotor Vigilance Task (PVT) [46], a software-based measure of reaction time (delivered using an iPad). Based on previous research [47,48], the following outcomes for PVT were included: mean reaction time, mean reciprocal reaction time (1/RT), fastest 10% reaction times, slowest 10% reciprocal reaction times, number of lapses (RT ≥ 500 ms), and false starts (RT < 100 ms). Controlled response was measured using the NBack [49], where participants need to determine whether a stimulus they see was the same as the one seen two image(s) before. Accuracy score, mean response times, and number of incorrect responses were measured for NBack [50,51].

In addition, data related to participants engagement with SleepSync was recorded using the app. This included information related to how many days participants logged on to the app and how many requests for recommendations were sent to the Alertness API. It must be noted that the requests for recommendations were sent to the API directly whenever participants added shifts or commute related information or added sleep information. We also asked participants questions related to their likelihood of using recommendations and what aspects of the app they found particularly useful or challenging. Please note that the information related to usefulness and challenging aspects of the app are not reported in the study as they form part of the future iteration and further development of the app.

### 4.5. Data Analyses

All data were analysed in R. Descriptive data are reported as mean and standard deviation (M  ±  SD). Data were analysed using non-parametric and semi-parametric approaches due to limited sample size. A power analysis based on a review of previous insomnia interventions for shift workers [12] suggested that a sample size of 21–24 per group is required to detect a large effect in ISI with 80% power at an alpha level of 0.05. Bonferroni corrections were applied to all analyses. For all questionnaire-based measures, the Wilcoxon signed rank test was used to examine differences prior to and during app use (alpha level set to 0.005). Exploratory analyses for objective measures to determine whether testing period (baseline vs during app use), shift type (morning vs evening vs night), and start or end of the shift were associated with changes in sustained attention and controlled response. For all PVT (alpha level set at 0.008) and NBack (alpha level set to 0.012), generalised additive mixed models (GAMM) were created using the MGCV package (https://cran.r-project.org/web/packages/mgcv/). GAMMs are semi-parametric tests that serve as an extension for mixed-effect modelling, with fixed effect terms (or factor variable) and random effects (smooth terms). They are a recommended technique when associations between predictor and outcome variables are not necessarily linear. For all models, participants were added as smooth to assess inter-individual differences. Estimated degrees of freedom (EDF) were calculated for each model to examine linearity of relationships between predictor(s) and outcome variables. For each GAMM, we first assessed the best-fitting model based on the Akaike information criterion (AIC), with baseline model consisting of only testing period. Additional factor variables (i.e., shift type—morning, evening, or night, start or end of the shift) were then added, and AIC compared. Adjusted R2 values are presented with each model to depict variance explained in outcome variables by each model. Spearman correlations, examining potential associations between changes in ISI during the study, and PVT and N-Back outcomes are presented in the Supplementary File.

## 5. Conclusions

Defence personnel often work in safety-critical, high-stress operations, which can result in sleep and circadian disruption. This early study shows that a personalised, app-based tool for sleep health support in shift working defence personnel may have positive impacts on their self-reported sleep. Findings from this study also demonstrate the utility of digital interventions in providing a confidential, scalable, and accessible pathway for delivering interventions for shift workers in general. Trials with a larger sample size, other cohorts of shift workers in defence with different work hours or schedules are required to establish the utility of this intervention across different settings. Randomised controlled trials, with longer follow-up periods assessing important operational and cognitive fitness outcomes are warranted to examine effectiveness and compliance with the intervention.

## Figures and Tables

**Table 1:** Summary of participant characteristics (n = 13).

Participant Characteristics	Mean ± SD or n (%)
Age (in years), mean ± SD range	29.28 ± 6.13 22–46
Sex, n (%) Female	6 (46.15)
Years of Experience (in years), mean ± SD range	5.61 ± 3.2 2–12
Education, n (%) Vocational or Diploma Bachelors Masters	7 (53.85) 4 (30.77) 2 (15.38)
Overnight Shifts, n (%) Yes	9 (69.23)
Dependents, mean ± SD range	0.74 (0.61) 0–2

**Table 2:** Generalised additive mixed models demonstrating differences for PVT outcomes from baseline to during app use.

	**Mean RT**				**Mean Reciprocal RT**				**Fastest 10% RT**			
**Variable**	**Estimates**	**95% CI**	**t-value**	***p***	**Estimates**	**95% CI**	**t-value**	***p***	**Estimates**	**95% CI**	**t-value**	***p***
Intercept	274.86	237.04–312.67		<0.001	3.74	3.51–3.98		<0.001	211.72	201.24–222.20		<0.001
**Testing period**												
Two weeks of app intervention	41.52	17.16–65.87	3.336	**0.001**	−0.03	−0.19–0.14	−0.29	0.768	2.63	−4.04–9.30	0.77	0.437
Four weeks of app intervention	35.89	10.30–61.48	2.77	**0.006**	0.01	−0.17–0.18	0.07	0.94	−7.45	−14.46–−0.44	−2.09	0.037
**Shift type**												
Evening shift	20.88	−2.42–44.18	1.77	0.079	−0.02	−0.19–0.14	−0.29	0.769	−0.47	−6.85–5.91	−0.14	0.885
Night shift	27.69	−8.88–64.25	1.5	0.137	−0.08	−0.33–0.17	−0.64	0.52	0.34	−9.68–10.36	0.06	0.947
**Start or end of shift**												
End of shift	15.89	−4.29–36.07	1.55	0.122	−0.12	−0.26–0.02	−1.71	0.088	5.85	0.33–11.38	2.09	0.038
**Smooth terms**												
Participant ID	**EDF**	**RefDF**	**F-value**		**EDF**	**RefDF**	**F-value**		**EDF**	**RefDF**	**F-value**	
	10.68	12	10.28	**<0.001**	10.23	12	6.59	**<0.001**	10.72	12	10.14	**<0.001**
**Adjusted R2**	0.47				0.31				0.42			
	**Slowest 10% Reciprocal RT**				**Lapses**				**False starts**			
**Variable**	**Estimates**	**95% CI**	**t-value**	***p***	**Estimates**	**95% CI**	**t-value**	***p***	**Estimates**	**95% CI**	**t-value**	***p***
Intercept	2.47	2.13–2.82		<0.001	4.98	3.58–6.38		<0.001	1.17	0.28–3.22	2.35	0.020
**Testing period**												
Two weeks of app intervention	−0.28	−0.46–−0.09	−2.97	**0.003**	−0.65	−1.81–0.51	−1.1	0.27	1.12	0.16–2.07	2.30	0.024
Four weeks of app intervention	−0.33	−0.53–−0.14	−3.41	**0.001**	−1.06	−2.27–0.16	−1.71	0.089	0.05	−0.96–1.06	0.09	0.920
**Shift type**												
Evening shift	−0.14	−0.32–0.04	−1.55	0.121	1	−0.10–2.10	1.79	0.075	0.60	−0.32–1.51	1.28	0.200
Night shift	−0.28	−0.56–−0.00	−1.99	0.047	1.1	−0.60–2.81	1.27	0.204	0.29	−1.15–1.72	0.39	0.693
**Start or end of shift**												
End of shift	−0.02	−0.17–0.13	−0.24	0.806	−0.03	−0.99–0.94	−0.05	0.956	0.37	−0.42–1.17	0.92	0.354
**Smooth terms**												
Participant ID	**EDF**	**RefDF**	**F-value**		**EDF**	**RefDF**	**F-value**		**EDF**	**RefDF**	**F-value**	
	11.18	12	17.07	**<0.001**	9.22	12	3.41	**<0.001**	10.64	12	11.77	**<0.001**
**Adjusted R2**	0.58				0.23				0.46			

Notes: Referent categories (used for intercept): Testing period: baseline, shift type: morning shift, start or end of the shift: start of the shift. Abbreviations: PVT = Psychomotor Vigilance Task, RT = reaction time. Total number of observations = 186 across 13 participants (Baseline = 67, two weeks of app intervention = 65, four weeks of app intervention = 57; shift distribution—morning shifts = 76, evening shifts = 68, night shifts = 42). While nine participants suggested having overnight shifts, only six participants recorded overnight shifts during the testing period. All other participants reported morning and evening shifts. Bonferroni correction applied; alpha value set to 0.008.

**Table 3:** Generalised additive mixed models demonstrating differences for N-Back outcomes from baseline to during app use.

	**NBack Mean RT**				**NBack Correct Responses**				**NBack Incorrect Responses**			
**Variable**	**Estimates**	**95% CI**	**t-value**	***p***	**Estimates**	**95% CI**	**t-value**	***p***	**Estimates**	**95% CI**	**t-value**	***p***
Intercept	627.7	585.13–670.27		<0.001	52.57	50.24–54.90		<0.001	9.36	7.48–11.24		<0.001
**Testing period**												
Two weeks of app intervention	−0.74	−30.16–28.68	−0.04	0.96	−0.34	−2.04–1.35	−0.39	0.691	0.23	−1.32–1.78	0.29	0.771
Four weeks of app intervention	−13.2	−43.93–17.52	−0.84	0.397	0.41	−1.36–2.18	0.45	0.649	−3.87	−5.49–−2.24	−4.7	**<0.001**
**Shift type**												
Evening shift	−7	−34.69–20.68	−0.49	0.618	0.47	−1.13–2.06	0.57	0.563	−0.14	−1.60–1.31	−0.19	0.848
Night shift	−28.67	−74.39–17.04	−1.23	0.217	1.01	−1.61–3.64	0.76	0.448	−0.85	−3.23–1.53	−0.7	0.481
**Start or end of shift**												
End of shift	11.28	−12.98–35.55	0.91	0.36	−0.07	−1.46–1.33	−0.09	0.926	−0.26	−1.54–1.03	−0.39	0.692
**Smooth terms**	**EDF**	**RefDF**	**F-value**		**EDF**	**RefDF**	**F-value**		**EDF**	**RefDF**	**F-value**	
Participant ID	10.43	12	7.93	**<0.001**	10.19	12	7.53	**<0.001**	9.38	12	4.33	**<0.001**
**Adjusted R2**	0.34				0.36				0.30			
	**NBack Accuracy Score**											
**Variable**	**Estimates**	**95% CI**	**t-value**	***p***								
Intercept	509.82	416.57–603.07		<0.001								
**Testing period**												
Two weeks of app intervention	19.64	−46.07–85.35	0.59	0.556								
Four weeks of app intervention	10.06	−58.00–78.13	0.29	0.771								
**Shift type**												
Evening shift	58.15	−3.29–119.59	1.86	0.063								
Night shift	64.16	−37.07–165.39	1.25	0.213								
**Start or end of shift**												
End of shift	30.33	−23.70–84.37	1.11	0.269								
**Smooth terms**	**EDF**	**RefDF**	**F-value**									
Participant ID	10.38	12	7.53	**<0.001**								
**Adjusted R2**	0.33											

Notes: Referent categories (used for intercept): testing period: baseline, shift type: morning shift, start or end of the shift: start of the shift. Abbreviations: RT = reaction time. Total number of observations = 183 across 13 participants (Baseline = 64, two weeks of app intervention = 65, four weeks of app intervention = 57; shift distribution—morning shifts = 74, evening shifts = 67, night shifts = 42). While nine participants suggested having overnight shifts, only six participants recorded overnight shifts during the testing period, all other participants reported morning and evening shifts. Bonferroni correction applied; alpha value set to 0.012.

## Data Availability

Data may be available from researchers upon reasonable request.

## Supplementary Materials

The following supporting information can be downloaded at: https://www.mdpi.com/article/10.3390/clockssleep6020019/s1. Table S1. Spearman and Intraclass correlations between ISI and a. PVT, b. N-Back.

## References

[1] Cooper A.D., Kolaja C.A., Markwald R.R., Jacobson I.G., Chinoy E.D. (2021). Longitudinal associations of military-related factors on self-reported sleep among U.S. service members. Sleep.

[2] Good C.H., Brager A.J., Capaldi V.F., Mysliwiec V. (2020). Sleep in the United States Military. Neuropsychopharmacology.

[3] Schmied E.A., Harrison E.M., Easterling A.P., Hurtado S.L., Glickman G.L. (2022). Circadian, light, and sleep skills program: Efficacy of a brief educational intervention for improving sleep and psychological health at sea. Sleep Health.

[4] Sletten T.L., Cappuccio F.P., Davidson A.J., Van Cauter E., Rajaratnam S.M.W., Scheer F.A.J.L. (2020). Health consequences of circadian disruption. Sleep.

[5] Rajaratnam S.M.W., E Howard M., Grunstein R.R. (2013). Sleep loss and circadian disruption in shift work: Health burden and management. Med. J. Aust..

[6] Bramoweth A.D., Germain A. (2013). Deployment-Related Insomnia in Military Personnel and Veterans. Curr. Psychiatry Rep..

[7] Markwald R.R., Carey F.R., Kolaja C.A., Jacobson I.G., Cooper A.D., Chinoy E.D. (2021). Prevalence and predictors of insomnia and sleep medication use in a large tri-service US military sample. Sleep Health.

[8] Devine J.K., Collen J., Choynowski J.J., Capaldi V. (2020). Sleep disturbances and predictors of nondeployability among active-duty Army soldiers: An odds ratio analysis of medical healthcare data from fiscal year 2018. Mil. Med. Res..

[9] Grier T., Dinkeloo E., Reynolds M., Jones B.H. (2020). Sleep duration and musculoskeletal injury incidence in physically active men and women: A study of U.S. Army Special Operation Forces soldiers. Sleep Health.

[10] Main L.C., McLoughlin L.T., Flanagan S.D., Canino M.C., Banks S. (2023). Monitoring cognitive function in the fatigued warfighter: A rapid review of cognitive biomarkers. J. Sci. Med. Sport.

[11] Aidman E. (2020). Cognitive Fitness Framework: Towards Assessing, Training and Augmenting Individual-Difference Factors Underpinning High-Performance Cognition. Front. Hum. Neurosci..

[12] Crowther M.E., Ferguson S.A., Vincent G.E., Reynolds A.C. (2021). Non-Pharmacological Interventions to Improve Chronic Disease Risk Factors and Sleep in Shift Workers: A Systematic Review and Meta-Analysis. Clocks Sleep.

[13] Murray J.M., Magee M., Giliberto E.S., Booker L.A., Tucker A.J., Galaska B., Sibenaller S.M., Baer S.A., Postnova S., Sondag T.A. (2023). Mobile app for personalized sleep–wake management for shift workers: A user testing trial. Digit. Health.

[14] Vetter C., Fischer D., Matera J.L., Roenneberg T. (2015). Aligning Work and Circadian Time in Shift Workers Improves Sleep and Reduces Circadian Disruption. Curr. Biol..

[15] Booker L.A., Sletten T.L., Barnes M., Alvaro P., Collins A., Chai-Coetzer C.L., McMahon M., Lockley S.W., Rajaratnam S.M., Howard M.E. (2021). The effectiveness of an individualized sleep and shift work education and coaching program to manage shift work disorder in nurses: A randomized controlled trial. J. Clin. Sleep Med..

[16] Chinoy E.D., Harris M.P., Kim M.J., Wang W., Duffy J.F. (2016). Scheduled evening sleep and enhanced lighting improve adaptation to night shift work in older adults. Occup. Environ. Med..

[17] Shriane A.E., Rigney G., A Ferguson S., Bin Y.S., E Vincent G. (2023). Healthy Sleep Practices for Shift Workers: Consensus Sleep Hygiene Guidelines Using a Delphi Methodology. Sleep.

[18] Postnova S. (2019). Sleep Modelling across Physiological Levels. Clocks Sleep.

[19] Abeysuriya R.G., Lockley S.W., Robinson P.A., Postnova S. (2018). A unified model of melatonin, 6-sulfatoxymelatonin, and sleep dynamics. J. Pineal Res..

[20] Phillips A.J.K., Robinson P.A. (2007). A Quantitative Model of Sleep-Wake Dynamics Based on the Physiology of the Brainstem Ascending Arousal System. J. Biol. Rhythms.

[21] Phillips A.J.K., Klerman E.B., Butler J.P. (2017). Modeling the adenosine system as a modulator of cognitive performance and sleep patterns during sleep restriction and recovery. PLoS Comput. Biol..

[22] Song Y.M., Choi S.J., Park S.H., Lee S.J., Joo E.Y., Kim J.K. (2023). A real-time, personalized sleep intervention using mathematical modeling and wearable devices. Sleep.

[23] Varma P., Postnova S., Phillips A.J., Knock S., Howard M.E., Rajaratnam S.M., Sletten T.L. (2023). Pilot feasibility testing of biomathematical model recommendations for personalising sleep timing in shift workers. J. Sleep Res..

[24] Aidman E. (2017). The cognitive fitness framework: A roadmap for systematic, evidence-based mental skills training and performance enhancement. J. Sci. Med. Sport.

[25] Booker L.A., Barnes M., Alvaro P., Collins A., Chai-Coetzer C.L., McMahon M., Lockley S.W., Rajaratnam S.M.W., Howard M.E., Sletten T.L. (2019). The role of sleep hygiene in the risk of Shift Work Disorder in nurses. Sleep.

[26] Hattatoglu D.G., Aydin S., Aydin C., Yildiz B.P. (2020). The Effect of Sleep Hygiene and Sleep Deterioration on Quality of Life in Shiftworking Healthcare Professionals. Noro Psikiyatr. Arsivi.

[27] Willis S.L., Tennstedt S.L., Marsiske M., Ball K., Elias J., Koepke K.M., Morris J.N., Rebok G.W., Unverzagt F.W., Stoddard A.M. (2006). Long-term Effects of Cognitive Training on Everyday Functional Outcomes in Older Adults. JAMA.

[28] Gardner B., Lally P., Wardle J. (2012). Making health habitual: The psychology of ‘habit-formation’ and general practice. Br. J. Gen. Pract..

[29] Mastin D.F., Bryson J., Corwyn R. (2006). Assessment of sleep hygiene using the Sleep Hygiene Index. J. Behav. Med..

[30] Ritland B.M., Simonelli G., Gentili R.J., Smith J.C., He X., Mantua J., Balkin T.J., Hatfield B.D. (2019). Effects of sleep extension on cognitive/motor performance and motivation in military tactical athletes. Sleep Med..

[31] Ingre M., Van Leeuwen W., Klemets T., Ullvetter C., Hough S., Kecklund G., Karlsson D., Åkerstedt T. (2014). Validating and Extending the Three Process Model of Alertness in Airline Operations. PLoS ONE.

[32] Riedy S.M., Fekedulegn D., Andrew M., Vila B., Dawson D., Violanti J. (2020). Generalizability of a biomathematical model of fatigue’s sleep predictions. Chronobiol. Int..

[33] Darwent D., Dawson D., Roach G.D. (2012). A model of shiftworker sleep/wake behaviour. Accid. Anal. Prev..

[34] Wright K.P., Gronfier C., Duffy J.F., Czeisler C.A. (2005). Intrinsic Period and Light Intensity Determine the Phase Relationship between Melatonin and Sleep in Humans. J. Biol. Rhythm..

[35] Stone J.E., Aubert X.L., Maass H., Phillips A.J.K., Magee M., Howard M.E., Lockley S.W., Rajaratnam S.M.W., Sletten T.L. (2019). Application of a Limit-Cycle Oscillator Model for Prediction of Circadian Phase in Rotating Night Shift Workers. Sci. Rep..

[36] Stone J.E., McGlashan E.M., Quin N., Skinner K., Stephenson J.J., Cain S.W., Phillips A.J.K. (2020). The Role of Light Sensitivity and Intrinsic Circadian Period in Predicting Individual Circadian Timing. J. Biol. Rhythm..

[37] Friedman M.J. (2006). Posttraumatic Stress Disorder Among Military Returnees From Afghanistan and Iraq. Am. J. Psychiatry.

[38] Reynolds A.C., Sweetman A., Crowther M.E., Paterson J.L., Scott H., Lechat B., Wanstall S.E., Brown B.W., Lovato N., Adams R.J. (2023). Is cognitive behavioral therapy for insomnia (CBTi) efficacious for treating insomnia symptoms in shift workers? A systematic review and meta-analysis. Sleep Med. Rev..

[39] Sletten T.L., Ftouni S., Nicholas C.L., Magee M., Grunstein R.R., Ferguson S., Kennaway D.J., O’brien D., Lockley S.W., Rajaratnam S.M.W. (2017). Randomised controlled trial of the efficacy of a blue-enriched light intervention to improve alertness and performance in night shift workers. Occup. Environ. Med..

[40] Slanger T.E., Gross J.V., Pinger A., Morfeld P., Bellinger M., Duhme A.-L., Ortega R.A.R., Costa G., Driscoll T.R., Foster R.G. (2016). Person-directed, non-pharmacological interventions for sleepiness at work and sleep disturbances caused by shift work. Cochrane Database Syst. Rev..

[41] Knock S.A., Magee M., Stone J.E., Ganesan S., Mulhall M.D., Lockley S.W., Howard M.E., Rajaratnam S.M.W., Sletten T.L., Postnova S. (2021). Prediction of shiftworker alertness, sleep, and circadian phase using a model of arousal dynamics constrained by shift schedules and light exposure. Sleep.

[42] Albertella L., Kirkham R., Adler A.B., Crampton J., Drummond S.P., Fogarty G.J., Gross J.J., Zaichkowsky L., Andersen J.P., Bartone P.T. (2023). Building a transdisciplinary expert consensus on the cognitive drivers of performance under pressure: An international multi-panel Delphi study. Front. Psychol..

[43] Morin C.M., Belleville G., Bélanger L., Ivers H. (2011). The Insomnia Severity Index: Psychometric Indicators to Detect Insomnia Cases and Evaluate Treatment Response. Sleep.

[44] Cella D., Yount S., Rothrock N., Gershon R., Cook K., Reeve B., Ader D., Fries J.F., Bruce B., Rose M. (2007). The Patient-Reported Outcomes Measurement Information System (PROMIS): Progress of an NIH Roadmap cooperative group during its first two years. Med. Care.

[45] Broomfield N.M., Espie C.A. (2005). Towards a valid, reliable measure of sleep effort. J. Sleep Res..

[46] Grant D.A., Honn K.A., Layton M.E., Riedy S.M., Van Dongen H.P. (2017). 3-minute smartphone-based and tablet-based psychomotor vigilance tests for the assessment of reduced alertness due to sleep deprivation. Behav. Res. Methods.

[47] Basner M., Mollicone D., Dinges D.F. (2011). Validity and Sensitivity of a Brief Psychomotor Vigilance Test (PVT-B) to Total and Partial Sleep Deprivation. Acta Astronaut..

[48] Basner M., Dinges D.F. (2011). Maximizing Sensitivity of the Psychomotor Vigilance Test (PVT) to Sleep Loss. Sleep.

[49] Kane M.J., Conway A.R.A., Miura T.K., Colflesh G.J.H. (2007). Working memory, attention control, and the N-back task: A question of construct validity. J. Exp. Psychol. Learn. Mem. Cogn..

[50] Armstrong N.C., Smith S.J.R., Risius D., Doyle D., Wardle S.L., Greeves J.P., House J.R., Tipton M., Lomax M. (2023). Cognitive performance of military men and women during prolonged load carriage. BMJ Mil Health.

[51] Meule A. (2017). Reporting and Interpreting Working Memory Performance in n-back Tasks. Front. Psychol..

