# Supplementary material for: SleepSync: Early Testing of a Personalised Sleep–Wake Management Smartphone Application for Improving Sleep and Cognitive Fitness in Defence Shift Workers
Source: Clocks Sleep. 2024 May 29;6(2):267–80. doi: 10.3390/clockssleep6020019 (PMC11203003; doi:10.3390/clockssleep6020019)
Supplement: Supplementary file 1 [file clockssleep-06-00019-s001.zip › clockssleep-2982647-supplementary.pdf]

Supplementary Table S1. Spearman and Intraclass correlations between ISI and a. PVT, b. N-Back.

**Variable**	**Spearman rho**	**95%CI**	***p***	**ICC**
**a. PVT**				
Mean RT	0.25	0.03 - 0.45	0.106	0.01
Mean Reciprocal RT	-0.22	-0.42--0.02	0.173	-0.06
Fastest 10% RT	0.31	0.10 - 0.48	**0.036**	0.04
Slowest 10% Reciprocal RT	0.22	0.01 - 0.42	0.103	0.00
False Starts	0.50	0.31 - 0.65	**0.002**	0.39
Lapses	0.30	0.10 - 0.44	0.058	0.24
**b. N-Back**				
Mean RT	0.13	-0.07 - 0.32	0.334	0.01
Correct Responses	-0.21	-0.03 - 0.01	0.243	-0.27
Incorrect Responses	0.40	0.21 - 0.55	**0.007**	0.49
Accuracy Score	-0.20	0.38 - -0.02	0.151	-0.01

Notes: Spearman's correlation used due to the non-parametric nature of these data. PVT And N-Back included repeated assessments, which was addressed in analyses using bootstrapping approach. Intraclass correlations suggest fair reliability for associations between ISI and PVT False Starts, ISI and N-Back Incorrect Responses.

Abbreviations: PVT – Psychomotor Vigilance Task; RT – Reaction Time.
